# Spatial variations in STIs among women enrolled in HIV prevention clinical trials in Durban, KwaZulu-Natal, South Africa

**DOI:** 10.1080/17290376.2023.2193238

**Published:** 2023-03-30

**Authors:** Reshmi Dassaye, Handan Wand, Tarylee Reddy, Frank Tanser, Benn Sartorius, Natashia Morris, Gita Ramjee

**Affiliations:** a HIV and other Infectious Diseases Research Unit, South African Medical Research Council, Durban, South Africa; b Kirby Institute, University of New South Wales, Kensington, Australia; c Biostatistics Unit, South African Medical Research Council, Durban, South Africa; d University of Lincoln, Lincoln Institute for Health, Lincoln, UK; e Faculty of Infectious and Tropical Diseases (ITD), London School of Hygiene & Tropical Medicine, London, UK; f Biostatistics Unit: GIS, South African Medical Research Council, Durban, South Africa; g London School of Hygiene and Tropical Medicine, Department of Epidemiology and Population Health, London, UK; h School of Medicine, Department of Global Health, University of Washington, Seattle, WA, USA; i The Aurum Institute, Johannesburg, South Africa

**Keywords:** STI, HIV, spatial epidemiology, mapping, incidence, South Africa

## Abstract

South Africa is faced with a high HIV and STI prevalence and incidence, respectively, with pockets of high burden areas driving these diseases. Localised monitoring of the HIV epidemic and STI endemic would enable more effective targeted prevention strategies. We assessed spatial variations in curable STI incidence among a cohort of women enrolled in HIV prevention clinical trials between 2002 and 2012. STI incidence rates from 7557 South African women enrolled in five HIV prevention trials were geo-mapped using participant household GPS coordinates. Age and period standardised incidence rates were calculated for 43 recruitment areas and Bayesian conditional autoregressive areal spatial regression (CAR) was used to identify significant patterns and spatial patterns of STI infections in recruitment communities. Overall age and period standardised STI incidence rate were estimated as 15 per 100 PY and ranged from 6 to 24 per 100 PY. We identified five significant STI high risk areas with higher-than-expected incidence of STIs located centrally (three-locations) and southern neighbouring areas of Durban (two-locations). Younger age (<25), not married/cohabitating, parity <3 and poor education were all significant correlates of high STI communities. Findings demonstrate sustained STI incidence rates across the greater Durban area. The role of STI incidence in HIV acquisition in high HIV endemic areas need to be revisited as current highly effective PrEP interventions do not protect from STI acquisition. In these settings there is an urgent need for integrative HIV and STI prevention and treatment services.

## Introduction

South Africa accounted for 200,000 new human immunodeficiency virus (HIV) infections in 2019 (UNAIDS, 2019) with nearly one million cases of other sexually transmitted infections (STIs) treated annually (SANC NSP, 2017–2022). High HIV and STI prevalence and incidence have been reported in the province of KwaZulu-Natal, South Africa (Johnson, Coetzee, & Dorrington, 2005; Nel et al., 2012), predominately among women (Frolich, Abdool Karim, Mashego, Sturm, & Abdool Karim, 2007; Hoque, 2011; Ramjee et al., 2005; Wilkinson, Connolly, Harrison, Lurie, & Abdool Karim, 1998). STIs are associated with enhanced HIV acquisition and transmission (Nagot et al., 2007; Patterson et al., 2008; Zhang et al., 2007) and therefore, potentially lead to poor sexual, reproductive and maternal child health (WHO, 2014; WHO, 2016). These findings coupled with high HIV incidence rates underscores the critical need to develop successful interventions to improve sexual and reproductive health in this region (Ramjee et al., 2019). In South Africa, STIs are managed syndromically using standardised national syndromic management guidelines (SA DOH, 2015). In 2017 the South African National Strategic Plan (NSP) for HIV, tuberculosis (TB) and STIs 2017–2022 aimed to employ geospatial mapping to identify high-risk populations (SANC NSP, 2017–2022). The spatial mapping of the clustering of infections at the local level, may provide insights about the drivers of these epidemics and will ultimately facilitate more efficient and effective use of resources and interventions.

The HIV Prevention Research Unit (HPRU) of the South African Medical Research Council (SAMRC) participated in five multi-centre HIV prevention trials at six sites across greater Durban in the province of KwaZulu Natal, from 2002–2012 (Abdool Karim et al., 2011; Marrazzo et al., 2015; McCormack et al., 2010; Padian et al., 2007; Skoler-Karpoff et al., 2008). Recently, we reported significant geographical variations in HIV infections in this region using the data from a cohort of women who enrolled in various biomedical prevention intervention trials (Ramjee et al., 2019).

The primary aim of this spatial analysis is to describe geographical variations in curable STI incidence (*Chlamydia trachomatis* (CT), *Neisseria gonorrhoea* (NG), syphilis and *Trichomonas vaginalis* (TV)) as observed in these trials over a period of 10 years. We also used Bayesian conditional autoregressive areal spatial regression (CAR) in order to identify significant geographical patterns of STIs. Bayesian hierarchical models are one of the main statistical techniques to investigate geographical variations. These models have been employed previously to provide fine-scale estimates of HIV prevalence in Sub-Saharan Africa and high-risk areas of TB/HIV coinfection in Kenya (Dwyer-Lindgren., 2019, Otiende et al., 2019). They are particularly useful to address the problems posed by small area analysis after accounting for the potential confounding effect of covariates as well as unmeasured heterogeneity within areas (Sartorius et al., 2013, 2020). Further, we examined the correlations between high STI risk areas, socio-demographic and sexual behaviours. In our recent publication we showed using the same dataset that within the generalised epidemic in Durban, there are pockets of high HIV incidence rates which may be further driving the epidemic (Ramjee et al., 2019). In the current analysis, we hypothesise a convergence of HIV- and spatial STI-hotspots in this region.

## Methods

### Study procedure and geographical area

From 2002 to 2012, the HPRU of the SAMRC has participated in five phase II/III multi-centre HIV prevention clinical trials (Abdool Karim et al., 2011; Marrazzo et al., 2015; McCormack et al., 2010; Padian et al., 2007; Skoler-Karpoff et al., 2008). A total of 9145 consenting women who enrolled in the respective trials were included in this combined cohort analysis and their places of residence (or nearest location point to residence) were geo-coordinated using Global Positioning Systems (GPS) at the time of enrolment and further updated during follow-up visits. This analysis includes GPS data from 7557 women. GPS coordinate data were collected using Garmin™ Nüvi (model 2360) handheld devices, downloaded into Microsoft Access and plotted spatially using ArcGIS (version 10.4, CA) (Redlands, 2011). A total of 43 community-level recruitment area boundaries were identified from the established census delineations (StatsSA, 2019).

Participants were recruited from communities in urban (peri-urban) and rural areas. A comprehensive description of the study population and procedures are described elsewhere (Abdool Karim et al., 2011; Marrazzo et al., 2015; McCormack et al., 2010; Padian et al., 2007; Skoler-Karpoff et al., 2008). For the current analysis, common data across the clinical trials was extracted, combined and reported as unidentified and not study or site-specific (Abdool Karim et al., 2011; Marrazzo et al., 2015; McCormack et al., 2010; Padian et al., 2007; Skoler-Karpoff et al., 2008). *P*-values less than 0.05 were considered statistically significant.

The age eligibility criteria were consistent across all trials (>18 years of age) except for one trial which enrolled women aged 16 and older. Median age across the trials varied marginally between 24 and 28 years. The average screening to enrolment ratio was 47% in this combined cohort. Other eligibility criteria were broadly similar for all studies. At each visit, participants received HIV risk reduction counselling, STI testing and treatment and had access to male and/or female condoms. Women who tested positive for an STI were provided with treatment according to the respective study protocols and local South African guidelines. Women who HIV seroconverted during the trial remained in the study and received ongoing safe sex counselling, STI testing and treatment, and condom provision. All participants provided written informed consent to participate in the studies.

### Laboratory procedures

HIV diagnostic testing was performed using two rapid tests on whole blood sourced from either finger-prick or venepuncture (Determine HIV-1/2, Abbot Laboratories, Tokyo, Japan and Oraquick, Orasure Technologies, Bethlehem, PA, USA). The Abbot IMX ELISA test (Abbot Diagnostics, Africa Division), in combination with the Vironostika HIV1/2 ELISA was used on whole blood sourced from venepuncture for discordant/unequivocal results.

The following methods were employed for STI detection depending on the clinical trial: (1) CT and GC was either detected by DNA PCR (Roche Pharmaceuticals, Branchburg, NJ, USA), PCR (COBAS Amplicor, Roche Molecular Diagnostics, Pleasanton, CA, USA) or the BDProbe Tec ET assay (Becton Dickinson, MD) using a urine sample, (2) TV was identified by PCR (Roche Pharmaceuticals, Branchburg, NJ, USA) or by wet mount microscopy and (3) syphilis was detected by rapid plasma re-agin (RPR) and confirmatory Treponema pallidum particle agglutination assay (TPHA) (Omega Diagnostics, Alva, UK) using whole blood sample.

### Socio-demographic data collection

The following characteristics were collected and combined across the five clinical trials: age, contraceptive use, STI at screening (CT, NG, syphilis and TV) and condom use at last sex act. Marital status/cohabiting and parity data were collected in four of the clinical trials. The number of sex partners in the last three months were collected in three of the five clinical trials.

### Characteristics of the study population

STI incidence rates were calculated in each of 15 strata (5 age groups for 3 time periods) in every recruitment area and mapped. Participant data were classified into five age categories (<20, 20–24, 25–29, 30–34, 35+ years old) across three-time periods (2002–2006, 2007–2009 and 2010–2012). We employed direct age-time period standardisation to obtain standardised STI incidence estimates per area to facilitate legitimate geographical comparison (free from the influence of underlying differences in the age-composition of participants in different areas as well as overall incidence changes over time).

**Figure 1.**
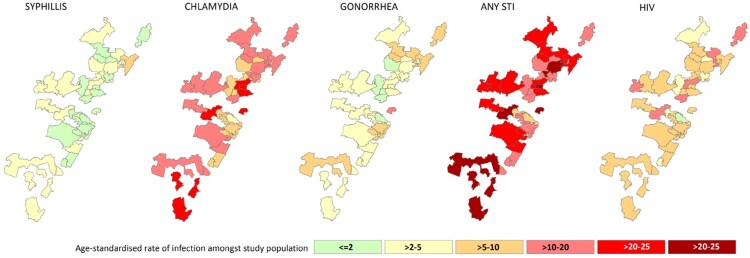
Age standardised STI and HIV rates.

The reference population considered for the age and period distribution were all women enrolled in the HIV prevention trials conducted at HPRU sites between 2002 and 2012. To ensure standardisation according to the same population type. An aggregate of women’s residence per recruitment area representing a minimum of 50 person years (PY) were included in STI incidence rate calculations (*n* = 7557) following exclusion of a) outlying participants not resident within core recruitment areas (*n* = 194), or residing in areas with less than 50 total PY (*n* = 207), (b) participants for whom no GPS data had been collected (*n* = 1054) and (c) participants who did not attend any follow up visits post enrolment (*n* = 133) (Figure 1). The crude STI incidence rate across all communities was calculated by dividing the number of new infections by total PY of observation. The supplementary figure 1, depicts the total number women who had STIs by clinical research sites.

The characteristics of the study population were described and formally compared across pre-defined STI incidence rate categories (≤10, 10–14 and 15+ per 100 PY). This analysis utilised data at the individual-level within this ordinal variable (i.e. categories of STI incidence rate). Age, marriage/cohabitation, type of contraception used, STI at baseline,

number of sexual partners in the last three months and parity data were included as the explanatory variables at the individual level. Univariable and multivariable ordered logistic regression, with recruitment area (community or group of communities) robust standard errors, were used to identify prominent factors associated with higher incidence. Stepwise forward selection regression (inclusion if *p* < 0.10) was used to construct the final multivariable model. Adjusted odds ratios (aOR) and 95% confidence intervals (CI’s) were presented in Table 2. The proportional odds (or parallel regression) assumption for this modelling approach was checked and upheld (Brant’s test) (Brant, 1990).

### Micro-geographical analysis

We employed a convolution conditional autoregressive (CAR) model proposed by Besag, York and Molliè (BYM) formulated as follows to assess incidence risk across the 43 areas (Besag, York, & Molliè, 1991):

$$O_{i}∼Poisson(E_{i}\gamma_{i}),\;\;\log(\gamma_{i})=\;\propto +\epsilon_{i}+w_{i}+\;{\text{β}}_{1}age_{i}+{\text{β}}_{2}period_{i}$$

where $O_{i}$ is the observed number of STIs in district i=1,…,43, $E_{i}$ is the expected number of STIs, $\gamma_{i}$ is the Poisson mean, ∝ is the constant, $\epsilon_{i}$ is the structured district heterogeneity random effect, $w_{i}$ is the conditional autoregressive (CAR) spatial term, ${\text{β}}_{1}$ and ${\text{β}}_{2}$ are the corresponding regression coefficients.

The spatially correlated random effect of the *i*th region (φiw_i_) is based on the sum of the weighted neighbourhood values and utilises an adjacency matrix of common neighbours (or shared boundaries) of a given area. For the unstructured recruitment area level random effect, we assumed an independent normal distribution ϵi ∼ N (0, σ2 ϵ) with variance σ2 ϵ. Non-informative gamma priors were used for both variance parameters, namely Gamma (0.5, 0.0005). We assumed vague normal distributions for the fixed effects coefficients (β). We estimated exceedance probability that risk ratio in a given recruitment area was significantly >1 and also utilised 95% uncertainty intervals around RRs. The model was fitted using Markov chain Monte Carlo simulation (Gilks, Richardson, & Spiegelhalter, 1996) and using WinBUGS software (Lunn, Thomas, Best, & Spiegelhalter, 2000). Model parameter posteriors were obtained once the Monte Carlo error for each parameter was less than 5% of the sample standard deviation.

### Ethics approval and consent to participate

Ethical approval for the trials were obtained from the University of KwaZulu-Natal Biomedical Research Ethics Committee and the South African Medical Research Council ethics committee (Ethics application numbers: T068/04, E165/04, T267/05, T101/04, EC08/1).

## Results

A total of 1253 incident STI infections observed in the total 8646 PY, corresponding to an overall crude incidence rate of 14.5 (13.7–15.3). Overall age and period standardised STI incidence rate were estimated as 15 per 100 PY and ranged from 6 Risecliff/Montford/Arena Park/Croftdene/to 24 per 100 PY in the Chesterville/Cato Manor/Mayville/Ridgeview. All crude and adjusted incidence rates are presented in Supplementary Table 1.

### Characteristics of the study population by STI incidence rates

Characteristics of areas were compared across increasing levels of STI incidence rates: <10 per 100 PY, 10–14 per 100 PY, 15+ per 100 PY (Table 1). There were significant differences observed between STI incidence categories and women’s characteristics. Compared to the areas with less than 10 and 10–14 per 100 PY, regions with 15+ STI incidence had significantly higher proportion of women younger than 25 years of age (44%, 41% vs 47%, respectively, *p* < 0.001). There was an increasing trend between the proportion of women who used injectables as a family planning method and STI incidence categories. Compared with women who were living in the areas with relatively lower STI incidence rates, those who resided where STI incidence rates were 15+, reported higher usage of injectables (46%, 51% vs 53%, respectively, *p* < 0.001). These areas had also significantly higher proportion of women with <3 children (88% vs. 79% and 83%, respectively, *p* < 0.001). In addition, 60% of the women who lived in the high STI incidence areas also reported that they don’t know whether or not their partner had another partner, compared to 52% and 54% where the incidence rates were <10 and 10–14 per 100 PY, respectively (*p* < 0.001). Further, a vast majority of the women were also reported to be unmarried and/or not cohabiting with their partners (89% vs 85% and 79%, respectively, *p* < 0.001). Women who lived in the highest HIV incidence rates were also correlated with the areas with high STI incidence rates.

**Table 1.** Characteristics of the study population by STI incidence rate.

		STI rates (age and time period standardised)			*p*-value
**Characteristics**	**Overall (*n* = 7.557)**	<10 per 100 PY (*n* = 853)	10–14 per 100 PY (*n* = 3.607)	15 + per 100 PY (*n* = 3.089)	
**Age (years)**					<0.001
<25 years	3.374 (45%)	345 (40%)	1.549 (43%)	1.480 (48%)	
25–29 years	1.602 (21%)	218 (26%)	722 (20%)	662 (21%)	
30 years	2.581 (34%)	290 (34%)	1.340 (37%)	951 (31%)	
**Marital Status**					<0.001
Married	1.564 (26%)	230 (30%)	821 (28%)	513 (22%)	
Not Married	4.467 (74%)	544 (70%)	2.065 (72%)	1.858 (78%)	
**Using injectables**					<0.001
No	3.577 (47%)	394 (46%)	1.814 (50%)	1.369 (44%)	
Yes	3.980 (53%)	459 (54%)	1.797 (50%)	1.724 (56%)	
**Using pills**					<0.001
No	6.773 (90%)	734 (86%)	3.261 (90%)	2.778 (90%)	
Yes	784 (10%)	119 (14%)	350 (10%)	315 (10%)	
**Parity**					<0.001
<3 children	4.715 (78%)	605 (78%)	2.184 (76%)	1.926 (81%)	
3+ children	1.317 (22%)	170 (22%)	703 (24%)	444 (19%)	
**Education**					<0.001
None	3.322 (55%)	249 (35%)	1.595 (58.25)	1.478 (56%)	
Primary	2.485 (41%)	437 (62%)	1.025 (37%)	1.023 (39%)	
Secondary higher	147 (6%)	16 (2%)	118 (6%)	147 (6%)	
**Partner has partner(excludes missing)**					<0.001
No	990 (25%)	169 (38%)	467 (24%)	354 (22%)	
Yes	734 (25%)	73 (16%)	369 (19%)	292 (18%)	
Don’t know	2.279 (57%)	204 (46%)	1.120 (57%)	955 (60%)	
**STI diagnosis (at baseline)**					<0.001
No	6.193 (82%)	742 (87%)	2.977 (83%)	2.474 (80%)	
Yes	1.356 (18%)	111 (13%)	630 (17%)	615 (20%)	
**Number of sex partners past 3m**					0.003
<3	3.929 (86%)	681 (88%)	1.835 (86%)	1.413 (83%)	
3+	661 (14%)	93 (12%)	286 (14%)	282 (17%)	
**HIV incidence rates**					<0.001
<5 per 100 PY	1.057 (14%)	292 (34%)	765 (21%)	–	
5–9 per 100 PY	5.723 (76%)	561 (66%)	2.530 (70%)	2.632 (85%)	
10+ per 100 PY	777 (10%)	–	316 (9%)	461 (15%)	

### Correlates of living in high STI areas

In the combined study population, overall age and period standardised STI incidence rate was estimated as 15 per 100 PY and ranged from 6 to 24 per 100 PY. Adjusted odds ratios (aORs) and 95% confidence intervals (CIs) were presented for the risk factors measured consistently across the trial populations (Table 2). In multivariable logistic regression model, compared to women who were married/living with their sexual partners those who were not married/not cohabiting with their sexual partners were significantly more likely to live in areas with STI incidence rates of 15 or more per 100 PY (aOR: 1.43, 95% CI: 1.19, 1.73, *p* < 0.001). Using non-barrier family planning methods such as injectables and oral contraceptives (i.e. pills) were also significantly more prevalent areas in with high STI incidence rates (aOR:1.15, 95% CI: 1.02, 1.29, *p* = 0.019 and aOR: 1.30, 95% CI: 1.07, 1.55, *p* = 0.006, respectively). Women who reported that they know their partner has another partner were also more common in STI high risk areas with aOR: 1.38 (95% CI: 1.16, 1.64, *p* < 0.001). In comparison, those who did not know whether their partner had another partner or not were also more significantly more prevalent in areas where incidence STI was estimated to be more than 15 per 100 PY (aOR: 1.30, 95% CI:1.04, 1.61, *p* = 0.020). Women who lived in these high STI incidence areas also had significantly low education (aOR: 1.25, 95% CI: 1.10, 1.42, *p* = 0.001). Although it was not statistically significant, we included baseline diagnosis with STI in the model because of its potentially confounding impact. Finally, we have observed significant correlation between the women who lived in high STI infections areas and high HIV prevalence areas. For example compared to women who lived in the regions with HIV incidence rates of <5, those who lived areas with 5–9 per 100 PY were 81% more likely to be the regions with highest STI infection rates (aOR: 1.81, 95% CI: 1.56, 2.11, *p* < 0.001); while women were more than four times more likely to be living in the high STI areas if they were coming from the regions where HIV incidence rate was estimated to be higher than 10 per 100 person year (aOR: 4.48, 95% CI: 3.62, 5.52, *p* < 0.001).

**Table 2.** Correlates of STI incidence rate 15+ per 100-person year.

Characteristics	Unadjusted Odds ratio (95% CI)	*p*-value	Adjusted Odds Ratio (95% CI)	*p*-value
**Age (years)**				
<25 years	1.28 (1.15, 1.44)	<0.001	–	
25–29 years	1.15 (1.01, 1.32)	0.049	–	
30+ years	1			
**Marital Status**				
Married	1		1	
Not Married	1.83 (1.54, 2.16)	<0.001	1.43 (1.19, 1.73)	<0.001
Missing	2.29 (1.90, 2.75)	<0.001	2.16 (1.74, 2.70)	<0.001
**Contraceptives**				
Others	1		1	
Injectables	1.22 (1.10, 1.36)	<0.001	1.15 (1.02, 1.29)	0.019
pills	1.25 (1.05, 1.48)	0.010	1.30 (1.07, 1.55)	0.006
**Parity**				
<3 children	1.60 (1.39, 1.85)	<0.001	1.20 (1.10, 1.42)	0.037
3+ children	1		1	
**Education**				
Some level	1		1	
Low education	1.35 (1.21, 1.52)	<0.001	1.25 (1.10, 1.42)	0.001
**Partner has partner(excludes missing)**				
No	1		1	
Yes	1.45 (1.23, 1.71)	<0.001	1.38 (1.16, 1.64)	<0.001
Don’t know	1.21 (0.98, 1.50)	0.078	1.30 (1.04, 1.61)	0.020
**STI diagnosis (at baseline)**				
No	1		1	
Yes	1.19 (1.04, 1.35)	0.010	1.13 (0.99, 1.29)	0.079
**HIV incidence rates**				
<5 per 100 PY	1		1	
5–9 per 100 PY	1.80 (1.55, 2.10)	<0.001	1.81 (1.56, 2.11)	<0.001
10+ per 100 PY	4.34 (3.53, 5.34)	<0.001	4.48 (3.62, 5.52)	<0.001
				

### Assessments of high STI incidence areas

**Figure 2.**
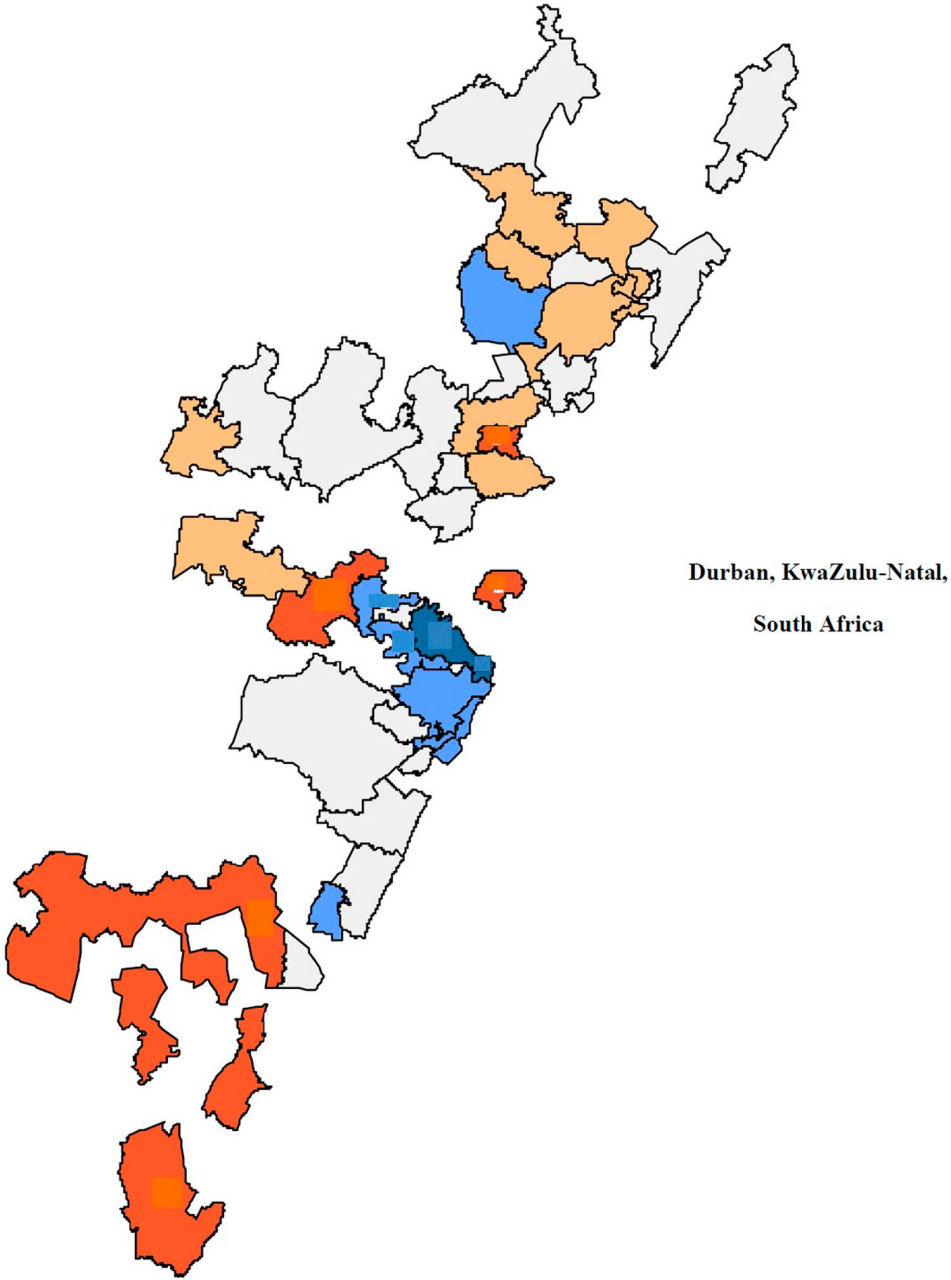
Formal identification of the STI incidence clusters.

Relative risks (RRs) from the Bayesian CAR model across the 43 communities are shown in Figure 2. The current analysis identified five high-risk areas located centrally (three) and southern neighbouring areas of Durban (two). Estimated RRs for these areas ranged from 1.31 to 1.5. Consistent with these results, age standardised STI incidence rates were also highest in these areas and estimated to be as high as 10–11 per 100 PY.

## Discussion

We observed a persistently high STI burden in the greater Durban area over a ten-year period; with STI incidence rates ranging from 6 to 24 per 100 PY. Similarly, a individual participant data (IPD) meta-analysis based on 18 prospective HIV prevention studies identified a higher STI prevalence in clinic/community-based populations in South Africa relative to homogenous populations elsewhere in Southern/Eastern Africa (Torrone et al., 2018). Additionally, an estimates study using the Spectrum-STI model (1990–2017) demonstrated a high incidence and prevalence of STIs in South Africa (Kularatne et al., 2018). Building on our finding of areas with high HIV infections in KwaZulu-Natal (Ramjee et al., 2019), in the current study, we identified five areas with significant risk of STI incidence; located centrally (three) and southern neighbouring areas of Durban (two). Also, we observed an overlap between some ‘high STI transmission areas’ and ‘high HIV transmission areas’ in this region. Other studies have also previously reported an overlap of HIV/STI prevalence and clustering (Hayes, Watson-Jones, Celum, van de Wijgert, & Wasser- heit, 2010).

The pathogenesis of HIV and STI, respectively share common risk factors including demographic, socio-economic characteristics and high sexual behaviours including condom-less sex (Zuma et al., 2005). The associations between the STIs and HIV infections have been well established (Kalichman, Pellowski, & Turner, 2011;; Mavedzenge et al., 2010 Wasserheit, 1992). Of concern is that the high STI incidence in the recruitment area in this study were observed among trial participants who received regular HIV pre- and post-test counselling, safe sex counselling, treatment of curable STIs and male and female condom promotion.

Interestingly, this study reported a high incidence of STIs among women using injectable or oral contraception. Previously, studies have suggested that use of the injectable DMPA may increase a woman’s HIV- risk, with meta-analyses finding a 40–50% increased HIV- risk (Morrison et al., 2015; Polis et al., 2016). The Evidence for Contraceptive Options and HIV. Outcomes (ECHO) Trial Consortium recently completed a randomised open-label clinical trial comparing contraception methods viz., intramuscular depot medroxyprogesterone acetate depot medroxyprogesterone acetate intramuscular (DMPA-IM), a copper intrauterine device (IUD) and a levonorgestrel (LNG) implant and HIV incidence (ECHO Consortium, 2019).

The ECHO trial was a pivotal study to ascertain the correlation between contraception use and HIV risk. The authors report no substantial difference in HIV risk among methods evaluated (ECHO Consortium, 2019). However, there was clear indication of the need to combine HIV prevention and reproductive health services in Africa.

Participants residing in areas with high STI incidence rates displayed the following characteristics: less than 25 years of age, not married/cohabitating, on injectables/oral contraceptives, did not know if partner had another partner and had a poor education. We previously demonstrated that characteristics including younger age (<25) and not being married/cohabitating were associated with high HIV and STI incidence rates (Ramjee et al., 2016; Ramjee & Wand, 2014). Francis et al., also demonstrated in a health and demographic surveillance site (HDSS) in rural KwaZulu-Natal, South Africa, a high prevalence of STIs and bacterial vaginosis among young men and women (aged 15–24 years old), respectively (Francis et al., 2018). In South Africa, other researchers also identified young age and not being married/cohabitating as risk factor for HIV/STIs (Rosenberg, Davidson, Chen, Judson, & Douglas, 1992; Rowley et al., 2019). This data emphasises the need for targeted health interventions for young adults.

According to the WHO, the total global estimated incident cases of chlamydia in 2016 was 127.2 million (95% UI: 95.1–165.9 million) (Rowley et al., 2019). Chlamydia infection has been linked to pelvic inflammatory disease, ectopic pregnancy, preterm labour, infertility, chronic pelvic pain and arthritis and can be transmitted during pregnancy and delivery to the newborn leading to conjunctivitis, trachoma and pneumonia (Rowley et al., 2019). In South Africa, the estimated female and male prevalence of chlamydia among adults aged 15–49 years was 14.7% (95% Cl: 9.9–21%) and 6.0 (95% Cl:3.8–10.4%), respectively, accounting for the highest estimated prevalence of the three curable STIs (viz.,.syphilis 0.50% (95% CI:0.32–0.80%) and 0.97% (0.19–2.28%), for gonorrhoea 6.6% (3.8–10.8%) and 3.5% (1.7–298 6.1%), respectively) (Kularatne et al., 2018). Further, we previously reported a high incidence of chlamydia 10.87 per 100 PY among pregnant participants enrolled in HIV-prevention clinical trials from 2002 to 2012 (Ramjee, Dassaye, Reddy, & Wand, 2018). Chlamydia infections are the most common STI worldwide and hence, chlamydia vaccine research has picked up stream. A recent phase 1 randomised, double-blind, placebo-controlled trial testing the chlamydia vaccine candidate CTH522 adjuvanted with CAF01 liposomes or aluminium hydroxide was shown to be safe and tolerable amongst participants, with CTH522:CAF01 demonstrating a better immunogenicity profile (Abraham et al., 2019).

While the combined data from multiple trials provides a unique opportunity to employ geospatial mapping to identify STI hotspots, several limitations need to be considered. Participants were recruited and enrolled from high risk HIV populations and thus, the findings may not be generalisable to all women in KwaZulu-Natal, South Africa. However, this large combined data set provides a snapshot of the epidemic over a decade in this region. The various clinical trials employed slight variations in STI testing methods for STI diagnosis, where some methods may have been more sensitive than others. Finally, information pertaining to partner infection status and resistant infections were not collected for any of the clinical trials. STIs especially gonorrhoea has become progressively resistant to antibiotics and in 2008 South Africa introduced monitoring of antimicrobial resistance in cultures (Kularatne, Maseko, Gumede, Readebe, & Kufa-Chakezha, 2016; Kularatne, Radebe, Kufa-Chakezha, Mbulawa, & Lewis, 2017). Further, South Africa is collaborating with the WHO Gonococcal Antimicrobial Surveillance Programme (GASP) (WHO, 2019). Intensification of actions to prevent, detect and control STIs alongside HIV are important if the burden of STIs is to be reduced. The WHO and several researchers have recognised one such intervention the development and availability of reliable, low-cost point of care diagnostics which provide diagnosis and treatment in a single visit (WHO, 2020).

## Conclusion

We identified high STI incidence across the greater Durban area in KwaZulu-Natal, South Africa, with an overlap among some ‘high STI transmission areas’ and ‘high HIV transmission areas’ (anonymous). Further, young women in this region are predominately at high risk of both HIV and STI acquisition. Identification of these ‘hot-spots’ is critical for targeted biomedical, behavioural and structural interventions to reduce the burden of HIV/STIs especially among the young women in these communities. Globally, there is an urgent need to address the high burden of STIs and its impact on HIV incidence and reproductive health.

## Data Availability

For the current analysis, common data across the clinical trials was extracted, combined and reported as unidentified and not study or site-specific. All data generated or analysed during this study are included in this published article [and its supplementary information files].
